# Promoting Access and Equity: A Historical Perspective of Healthcare Access for People With Disabilities

**DOI:** 10.7759/cureus.31594

**Published:** 2022-11-16

**Authors:** Jason M Rotoli, Anika Backster, Cori Poffenberger, Wendy C Coates

**Affiliations:** 1 Emergency Medicine, University of Rochester, Rochester, USA; 2 Emergency Medicine, Emory University School of Medicine, Atlanta, USA; 3 Emergency Medicine, University of New Mexico, Albuquerque, USA; 4 Emergency Medicine, David Geffen School of Medicine at UCLA (University of California Los Angeles), Los Angeles, USA

**Keywords:** ableism, mobility limitation, blindness, deafness, americans with disabilities act, accommodations, provider education and collaboration, access to health care, disability, disability advocacy

## Abstract

People with disabilities represent a large and often under-recognized minority population in the United States. Historically, negative healthcare provider perceptions and limited critical social determinants of health (including community living and education) have resulted in inequitable healthcare and access for this vulnerable group. Within the last 40 years, there have been some advances in legislation to improve access and support for those with disabilities. Since then, advances in accommodations have enabled better access to critical health-related resources and care. Continued forward progress and increased awareness are imperative to improve access, reduce disparities in healthcare, and combat discrimination.

## Editorial

Historical perspective

People with disabilities represent the largest minority population in the United States, with one in five people identifying as having a disability. Historically, people with disabilities have been marginalized and experienced “othering” (judgments of superiority and inferiority due to differences between groups leading to oppression) because of their minority status [1].

Marginalization mirrors the medical perspective of disease to identify the abnormality and treat it to return to “normal.” This reinforces ableism: the concept that able-bodied people represent the “perfect-self” or perfection of the human species [1].

Physicians may experience negative feelings about the disability community. This bias may undermine the physician-patient relationship, limit the ability to obtain relevant information, and negatively impact care for patients with disabilities [2]. People with disabilities are more likely to rate their health as poor, have higher rates of obesity and smoking, underutilize primary care services, and are more impacted by social determinants of health than the general population [3]. These multifactorial health disparities arise from structural and systematic barriers to healthcare (lack of access to care and awareness of the needs of the disability community, inadequate provider education, and bias rooted within the history of medicine and society) [1,4].

Evolving perspective

The language used to describe individuals with disabilities has evolved since acknowledging the ableist roots of historical terms which inappropriately medicalized disabilities and emphasized dependency (e.g., “suffers from,” “wheelchair-bound,” “handicapped,” “mentally retarded”) rather than utilizing neutral, descriptive terms such as “physical disability” or “intellectual disability.” In 2010, President Obama signed a bill that replaced the terms “mental retardation” and “mentally retarded" from federal records with "intellectual disability" and "individual with an intellectual disability."

Some advocate for the use of person-first language, a “person with a disability” rather than a “disabled person.” However, others prefer identity-first language to convey that their disability is inseparable from their identity, not something they “have.” Those favoring the term “disabled person” argue that they are disabled by surrounding social structures, rather than “having” a disability. It is best to ask about individual preferences. To circumvent the word “disability,” euphemisms such as “differently abled,” “handi-capable,” or “special needs” were conceived. These terms promote othering by implying that a “disability” is inherently negative. Thus, it is advisable to use the word “disability” instead of euphemisms.

In the 1960s-70s, the disability-rights movement enacted changes in policies promoting inclusion and community-based support, including the passage of the Rehabilitation Act of 1973 (amended 1978) and the Education for All Handicapped Children Act in 1975 (renamed The Individuals with Disabilities Act, “IDEA”) which fostered independent community living and public education, respectively. In 1990, the Americans with Disabilities Act (ADA) mandated inclusion/accommodation and barred discrimination toward people with disabilities in all areas of public life, including healthcare. Titles II (state and local governments) and III (private businesses and non-profit organizations) require that medical providers afford individuals with disabilities full and equal access to healthcare services and facilities, including making reasonable modifications to policies, practices, and procedures to allow the same access as the general population.

While intended to provide advocacy and a voice to those who are underrepresented, the efficacy of the ADA remains questionable. Employers may be unwilling to hire employees with disabilities for fear of increased financial burden. Healthcare organizations frequently place the cost of providing accommodations on single departments or individual clinicians’ offices, creating negative incentives for providing appropriate care and access for patients with disabilities.

Advances in accommodations

With the passage of the ADA, there has been a shift toward creating a more equitable environment by improving access to accommodations for those with disabilities. Prior to the ADA, there was no federal mandate for auxiliary aids or language services for blind or non-verbal patients, nor language accommodations for Deaf American Sign Language (ASL) users or deaf/hard-of-hearing individuals requiring transcription services. Some organizations provided no accommodations or a “one-size-fits-all” solution that did not meet individual needs, such as an ASL interpreter assigned to an oral deaf person who doesn’t understand ASL. This precluded interaction between patient and provider infringed on the patient’s right to healthcare and perpetuated lower levels of health literacy among patients requiring language accommodations. The ADA increased access to a variety of communication accommodations engendering more equitable care.

The first recognized accessible mobility design was published in 1961, was federally recognized in 1974, and was incorporated into the ADA in 1990. In 2017, an update specifically related to hospital medical equipment was added. ADA’s Title III required improved access to buildings (automatic doors, ramps, obstacle-free pathways) and doors/hallways and treatment rooms large enough to accommodate persons in wheelchairs.

To shift provider education, behavior, and attitudes, the World Health Organization published an integrated model of disability that shifted attention away from physical differences emphasized in the archaic medical model of disability and described bodily limitations or impairments in the context of the surrounding environment emphasizing the social model of disability [5].

The Rehabilitation Act (1973) created the National Institute on Disability, Independent Living, and Rehabilitation Research to understand the independent living needs of people with disabilities and a nationally-funded network of Centers for Independent Living to support community living and independence for people with disabilities, providing resources and support for community integration.

The use of animals as a disability aid appeared in frescoes in 1 CE. Trained dogs assisted people with visual impairments in the 1750s in Paris. US legal protection for the use of animals as a disability aid did not occur until the 1920s and was formally recognized in 1990. Titles II and III of the ADA support the use of service animals in all public areas. Additionally, staff may ask two questions: (Q1) “Is the dog a service animal required because of a disability?” and (Q2) “What work or task has the dog been trained to perform?”

Despite federal and state mandates regarding inclusive language, appropriate communication, and mobility accommodations for people with disabilities, equitable access to education, employment, and healthcare remains challenging due to an infrastructure that is not designed to provide accommodations, nor promote understanding of unique circumstances. Patients with disabilities struggle to arrange transportation must remind office staff of accommodations needed prior to appointments, and re-educate staff and providers on their disability. They may inappropriately be viewed as inflexible when appointments are changed at the last minute or if accommodations are unavailable upon arrival.

People with disabilities and those requiring accommodations represent a sizable, heterogenous, and marginalized population. With the implementation of the ADA and other legislative changes, some advances have begun. Continued progress is imperative to improve access, combat discrimination, and reduce disparities in healthcare. Through increased awareness and education, each member of the healthcare team can reduce the prevalence of ableism, avoid othering, and provide an inclusive environment for people with disabilities.
